# Basic, translational, and clinical research – a short reflection

**DOI:** 10.20945/2359-4292-2024-0400

**Published:** 2024-11-06

**Authors:** Peter A. Kopp

**Affiliations:** 1 UNI Division of Endocrinology, Diabetology and Metabolism Lausanne Switzerland UNI – Division of Endocrinology, Diabetology and Metabolism Lausanne, Vaud Switzerland

Research in endocrinology, like in all other medical disciplines, covers a broad continuum including basic science, translational and clinical research. Translational research aims at bridging the gap between scientific science, clinical, epidemiological and population studies into practical clinical applications. While translational research is often conceptualized as a unidirectional process from "*bench to bedside*" or "*bedside to bench*", it is not a unidirectional process but a dynamic and cyclical approach. Importantly, the borders between the various approaches are not always clearly defined; rather, they should be viewed as part of a spectrum.

The field of endocrinology is particularly well suited to illustrate the dynamic interactions between the different types of research. For example, the critical role of the pancreas in the pathophysiology of diabetes mellitus was discovered by removing the organ from dogs (1). Then, in 1921, Frederick Banting and Charles Best successfully isolated insulin from canine pancreas tissue (1). This groundbreaking work would be best defined as "basic research". It was followed by the first successful treatment of a human patient with diabetes mellitus in 1922 (1), hence "translational research". Further work was needed to develop purification methods for insulin to make it suitable for larger scale use. This led to the first clinical research trials and widespread treatment of diabetes patients with insulin. Modifications of the insulin preparations in the 1930s then led to longer-acting insulin preparations, improving treatment regimens.

Basic science efforts then unraveled the amino acid sequence of insulin in the 1950s by Frederick Sanger^1^ (Nobel Prize in Chemistry in 1958) (1,2). The insulin gene was cloned in the late 1970s and early 1980, ultimately leading to the identification and characterization of its cDNA. This paved the way for recombinant insulin production replacing animal-derived insulins beginning in the 1980s (3). The clinical significance of this basic research, or the *translation* into the clinical practice, is obvious. But it did not stop there. Modifications of the insulin cDNA have led to the development of insulin analogs with altered pharmacokinetic properties that are rapid- or long-acting, significantly improving glycemic control (3).

Numerous other examples for all endocrine organs could be listed to illustrate the intricate interactions between the discovery of a hormone, its chemical characterization and synthesis, to clinical applications for hormone replacement.

Other examples of cross-fertilization between basic and clinical research and applications include the identification of inhibitors of hormone synthesis or hormone action for the treatment of numerous disorders. For example, selective estrogen receptor modulator (SERM) tamoxifen, through blocking the estrogen receptor alpha (ERα), plays a key role in the treatment of breast cancer (4). In the realm of endocrine carcinomas, specific inhibitors of receptors or intracellular kinases have fundamentally changed the therapeutic options (5).

These selected examples also underscore that research should not be confined narrowly by funding bodies. Basic research often lays the groundwork for future applications in unpredictable ways, and overemphasis on applied research with obvious societal relevance can stifle creative novel approaches (6).

This special issue of the *Archives of Endocrinology and Metabolism* is dedicated to topics covering both basic and translational endocrinology. The content of the included studies illustrates the the symbiotic relationship between these research domains, and that the synergy between basic, translational, and clinical research is crucial for fostering creative innovations that ultimately result in improving clinical care.

## Footnotes

1 Sanger won a second Nobel Prize in Chemistry in 1980 along with Walter Gilbert and Paul Berg for their contributions to DNA sequencing (Sanger method).
